# Spleen proteomics data from high fat diet fed mice

**DOI:** 10.1016/j.dib.2020.106110

**Published:** 2020-08-01

**Authors:** Sara L Svahn, Bagmi Pattanaik, Louise Grahnemo, Saray Gutierrez, Intawat Nookaew, John-Olov Jansson, Maria E. Johansson

**Affiliations:** a Dept. of Physiology, Institute of Neuroscience and Physiology, Gothenburg, Sweden; b Centre for Bone and Arthritis Research, Department of Internal Medicine and Clinical Nutrition, Institute of Medicine, The Sahlgrenska Academy, University of Gothenburg, Gothenburg, Sweden; c Dept. of Biology and Biological Engineering, Chalmers University of Technology, Gothenburg, Sweden; d Department of Biomedical Informatics, University of Arkansas for Medical Sciences, Little Rock, AR, United States

**Keywords:** Diet, High fat diet, Low fat diet, Saturated fatty acids, Polyunsaturated fatty acids, iTRAQ, Proteomics

## Abstract

The composition of the diet affects many processes in the body, including body weight and endocrine system. We have previously shown that dietary fat also affects the immune system. Mice fed high fat diet rich in polyunsaturated fatty acids survive *S. aureus* infection to a much greater extent than mice fed high fat diet rich in saturated fatty acids. Here we present data regarding the dietary effects on protein expression in spleen from mice fed three different diets, I) low fat/chow diet (LFD, *n* = 4), II) high fat diet rich in saturated fatty acids (HFD-S, *n* = 4) and III) high fat diet rich in polyunsaturated fatty acids (HFD-P, *n* = 4). We performed mass spectrophotometry based quantitative proteomics analysis of isolated spleen by implementing the isobaric tags for relative and absolute quantification (iTRAQ) approach. Mass spectrometry data were analyzed using Proteome Discoverer 2.4 software using the search engine mascot against *Mus musculus* in SwissProt. 924 proteins are identified in all sets (*n* = 4) for different dietary effects taken for statistical analysis using Qlucore Omics Explorer software. Only 20 proteins were found to be differentially expressed with a cut-off value of false discovery rate < 0.1 (*q*-value) when comparing HFD-S and HFD-P but no differentially expressed proteins were found when LFD was compared with HFD-P or HFD-S. The identified proteins and statistical analysis comparing HFD-S and HFD-P diets are available as a supplementary file S1. We identified a subset of proteins that showed an inverse expression pattern between two high fat diets. These differentially expressed proteins were further classified by gene ontology for their role in biological processes and molecular functions. Mass spectrometry raw data are also available via ProteomeXchange with identifier PXD020365.

**Specifications Table**

**Table:** 

Subject	Endocrinology, Diabetes, and Metabolism
Specific subject area	dietary proteomics
Type of data	Raw data, Excel files, Tables, Figures
How data were acquired	Samples were analyzed on an LTQ-Orbitrap Velos mass spectrometer interfaced with an Easy-nLC (Thermo Fisher Scientific). For relative quantification, the MS raw data files for each iTRAQ set were merged in the search using Proteome Discoverer version 2.4 (Thermo Fisher Scientific). The database search was performed with the Mascot search engine (Matrix Science) against *Mus musculus* in SwissProt version July 2019 (Swiss Institute of Bioinformatics, Switzerland).
Data format	Analyzed Raw
Parameters for data collection	C57BL/6 mice were fed one of three different diets for 8 weeks, I) low fat/chow diet (LFD), II) high fat diet rich in saturated fatty acids (HFD-S), or III) high fat diet rich in polyunsaturated fatty acids (HFD-P). Proteins from spleen were extracted and analyzed using quantitative proteomics. *n* = 4 per group.
Description of data collection	The experimental layout is presented in Fig. 1. Data presented in this manuscript are spleen proteins affected by three different diets, I) low fat/chow diet (LFD), II) high fat diet rich in saturated fatty acids (HFD-S), or III) high fat diet rich in polyunsaturated fatty acids (HFD-P) (Table 1). We identified 924 proteins present in all datasets of different dietary conditions (Supplementary file S1). With a cut-off value of false discovery rate < 0.1 (*q*-value), the number of differentially expressed proteins data is presented in a Venn diagram (Fig. 2), and Table 2 and the two high fat diets are visualized in a heat map (Fig. 3). 20 differentially expressed proteins were further classified by gene ontology (Fig 4).
Data source location	Gothenburg, Sweden
Data accessibility	Analyzed data are available within the article and raw mass spectrometric proteomics data have been deposited to the ProteomeXchange Consortium via the PRIDE [1] partner repository with the dataset identifier PXD020365.

## Value of the data

• These data present the dietary effects of different high fat diets on protein expression in the spleen and highlight the influence of fatty acid composition on protein expression in the spleen.
• Researchers interested in how a high fat diet affects protein expression will find these data to be a valuable resource.
• Using the same high fat diets, these data provide further insights based on previous studies showing an immunological shift in gene expression after a high fat diet rich in polyunsaturated fatty acids.
• These data can generate a hypothesis for new studies investigating the dietary effects on protein expression in other tissues, especially other immune tissues.

## 1 Data description

**Fig. 1:**
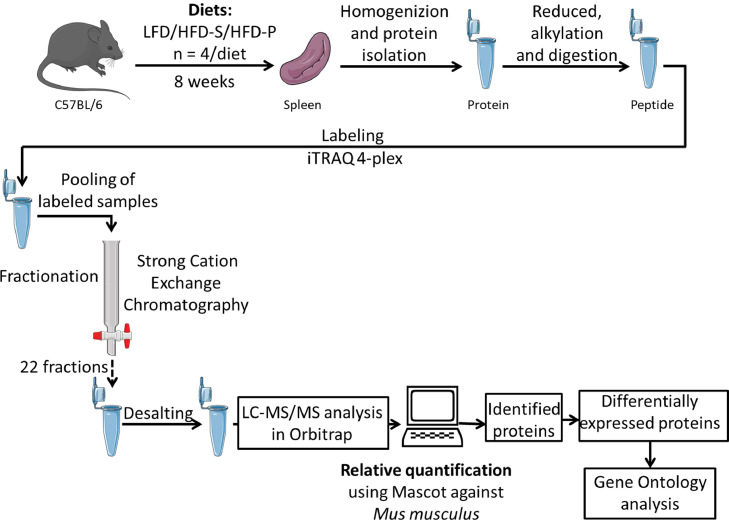
Experimental layout. C57BL/6 mice were fed low fat diet/chow diet (LFD), high fat diet rich in saturated fatty acids (HFD-S), or high fat diet rich in polyunsaturated fatty acids (HFD-P) for 8 weeks. Thereafter the spleens were harvested, processed, and analyzed.

**Fig. 2:**
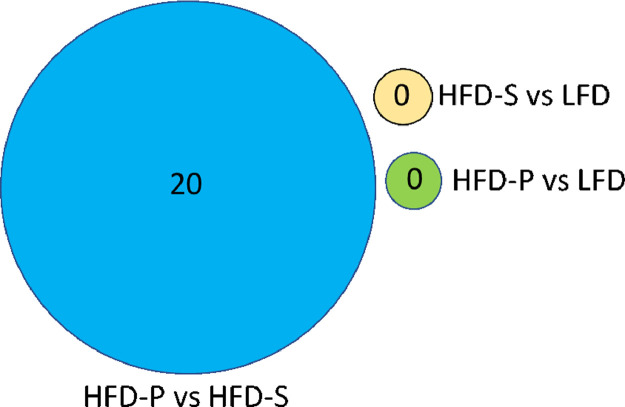
Venn diagram illustrating the number of differentially expressed proteins (false discovery rate < 0.1 (*q*-value)) from HFD-P vs HPD-S and no differentially expressed proteins identified with HFD-P vs LFD and HFD-S vs LFD.

**Fig. 3:**
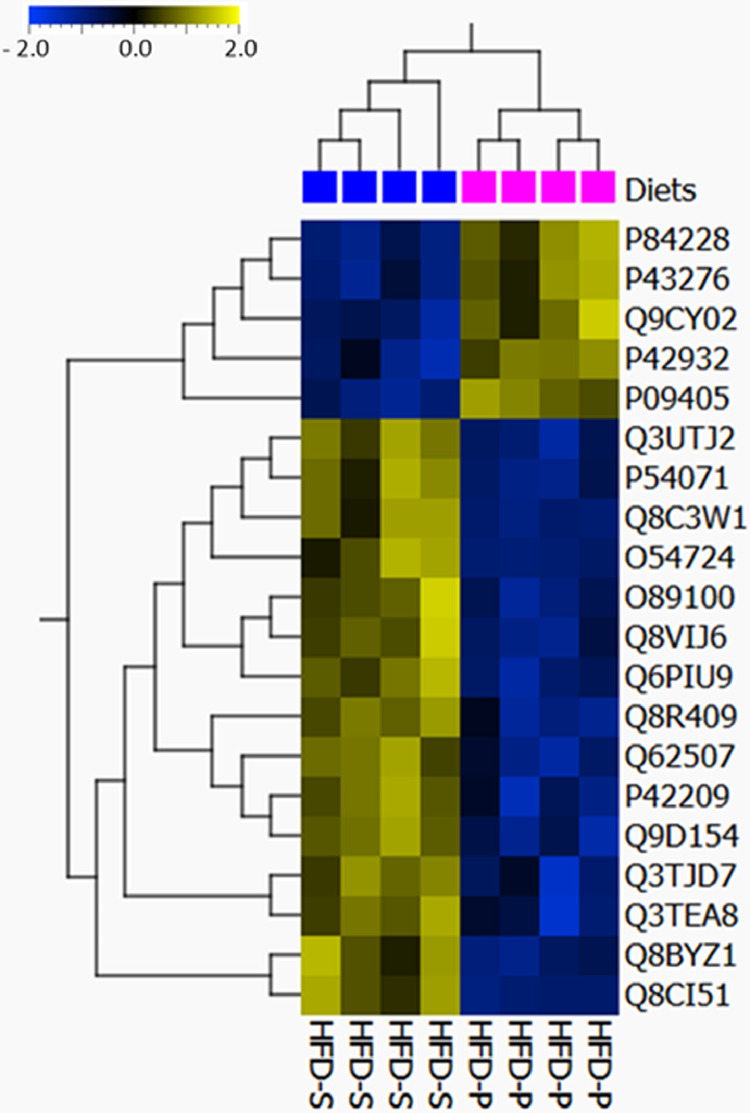
Heat map of hierarchical clustering shows 20 differentially expressed proteins (false discovery rate < 0.1 (*q*-value)) from mice spleen fed either a high fat diet rich in saturated fatty acids (HFD-S) or a high fat diet rich in polyunsaturated fatty acids (HFD-P) shown as a heatmap. Heatmap was created using Qlucore.

**Fig. 4:**
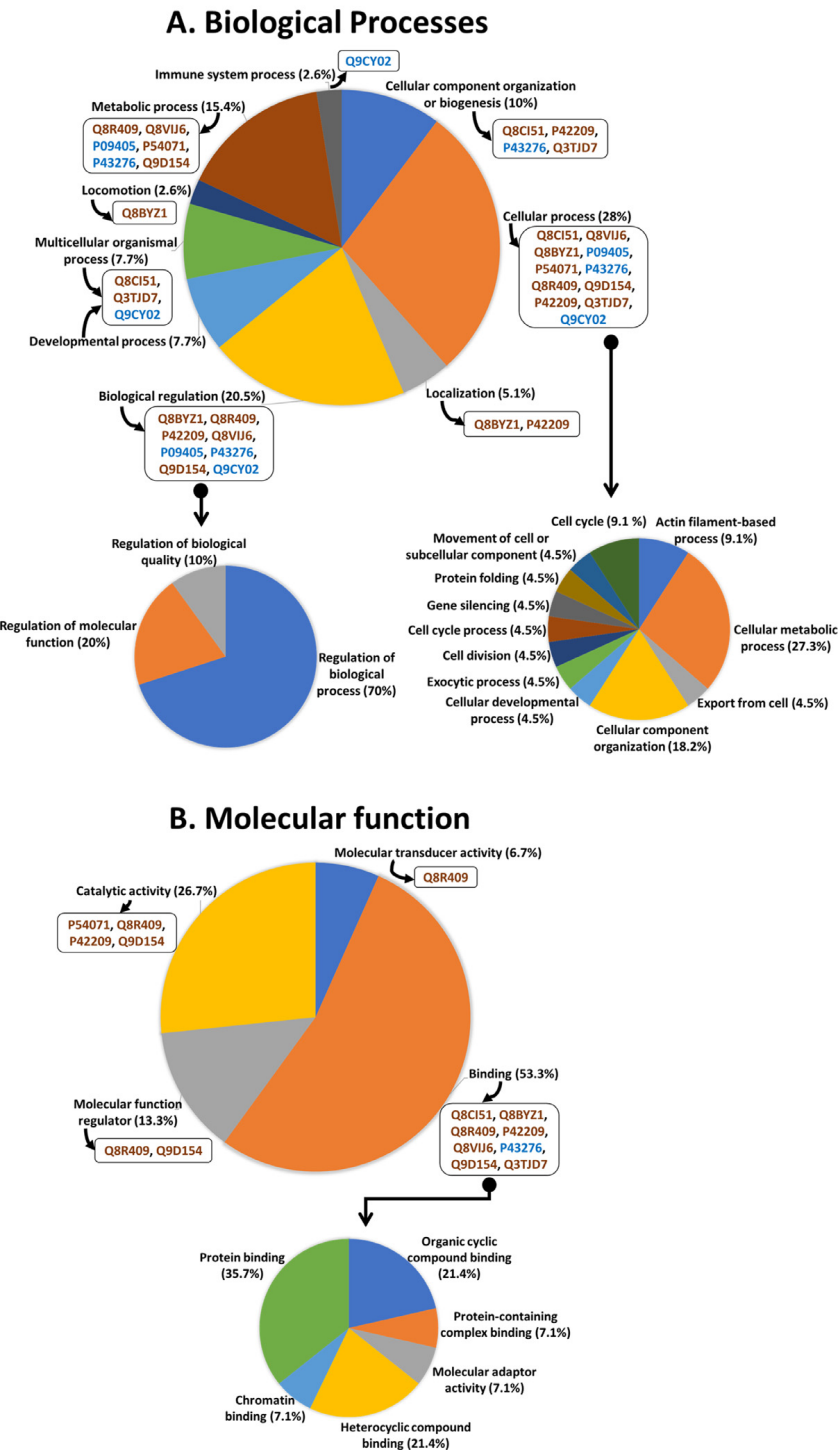
Functional analysis of the identified proteins involved in response to dietary fatty acid composition in mouse spleen using the PANTHER database. Pie chart representation of A) Biological processes and B) Molecular function. Specific proteins involved in the respective sub-processes are denoted in the box. Proteins in blue color represent down-regulated and orange color represents up-regulated in HFD-S compared with HFD-P. Major processes and functions identified were further analyzed for their sub-class categorization, presented in the smaller pie charts.

We used a proteomics approach to investigate the protein expression in the spleen after feeding mice a high fat diet with different fatty acid compositions. Fig. 1 provides the experimental layout. C57BL/6 mice were fed either I) low fat/chow diet (LFD), II) high fat diet rich in saturated fatty acids (HFD-S), or III) high fat diet rich in polyunsaturated fatty acids (HFD-P) (detailed composition is listed in Table 1) for 8 weeks. To identify the proteins expressed in the spleen in response to different diets, we carried out mass spectrophotometry based quantitative proteomics analysis of isolated spleen by implementing the isobaric tags for relative and absolute quantification (iTRAQ) approach. Extracted proteins from the spleen were further processed for proteomic analysis. The 924 proteins identified in all sets (*n* = 4) of different dietary conditions are listed in Supplementary file S1. With a cut-off false discovery rate < 0.1 (*q*-value), we identified 20 proteins that were differentially expressed between mice fed HFD-S and HFD-P and whereas there were no differentially expressed proteins identified in mice fed LFD compared with mice fed HFD-S or mice fed LFD compared with mice fed HFD-P (Fig. 2). An inverse protein expression pattern was identified between mice fed HFD-S and mice fed HFD-P (Fig. 3). Table 2 lists protein ID and name for the proteins included in Fig. 3. These differentially expressed proteins were further classified by gene ontology for their role in biological processes and molecular functions (Fig. 4).

**Table 1:** Energy density and composition of experimental diets: low fat/chow diet (LFD), high fat diet rich in saturated fatty acids (HFD-S), or high fat diet rich in polyunsaturated fatty acids (HFD-P). From Svahn SL et al [2,3].

	LFD	HFD-S	HFD-P
Energy density (kcal/g)	3.9	5.2	5.2
Macronutrients (% kcal)			
Protein	20	20	20
Carbohydrate	70	20	20
Fat	10	60	60
Fat source (% of total fat)			
Soybean oil	55.6	9.3	9.3
Lard	44.4	90.7	27.8
Menhaden oil	-	-	63.0
Fatty acids (% by wt of total fatty acids)			
∑ SFA	22.7	32.0	28.7
∑ MUFA	29.8	36.0	27.5
∑ PUFA	47.5	32.0	43.9
∑ n-3 total fat	5.2	2.1	25.6
∑ n-6 total fat	42.4	29.9	16.2
n-6/n-3	8.2	14.1	0.6

SFA, saturated fatty acids, MUFA; monounsaturated fatty acids; PUFA, polyunsaturated fatty acids; n-3, omega-3 fatty acid; n-6, omega-6 fatty acids.

**Table 2:** Protein ID and respective protein names presented in the heat map.

Protein ID	Protein name
Q9CY02	Alpha-hemoglobin-stabilizing protein
P84228	Histone H3,2
P43276	Histone H1,5
P09405	Nucleolin
P42932	T-complex protein 1 subunit theta
P54071	Isocitrate dehydrogenase [NADP], mitochondrial
Q8VIJ6	Splicing factor, proline- and glutamine-rich
Q3TEA8	Heterochromatin protein 1-binding protein 3
O89100	GRB2-related adaptor protein 2
Q3UTJ2	Sorbin and SH3 domain-containing protein 2
Q8R409	Protein HEXIM1
P42209	Septin-1
Q8BYZ1	ABI gene family member 3
Q6PIU9	Uncharacterized protein FLJ45252 homolog
Q8CI51	PDZ and LIM domain protein 5
Q62507	Cochlin
Q3TJD7	PDZ and LIM domain protein 7
Q9D154	Leukocyte elastase inhibitor A
Q8C3W1	Uncharacterized protein C1orf198 homolog
O54724	Caveolae-associated protein 1

## 2 Experimental design, materials and methods

Six-week-old male C57BL/6 mice were obtained from Harlan Netherlands B.V. (Horst, The Netherlands). The mice were housed under standard conditions of light and temperature at the animal facility at the Laboratory for Experimental Biomedicine, University of Gothenburg, Gothenburg, Sweden. Water and food were provided *ad libitum*. The regional ethical committee at Gothenburg University approved the experiments before the studies started.

### 2.1 Diets

At seven weeks of age, the mice were randomized into dietary-groups, which received one of the following diets: LFD (D12450B; 3.9 kcal/g, 10 kcal% fat, 20 kcal% protein, 70 kcal% carbohydrate; Research Diets, New Brunswick, NJ, USA), HFD-S (D12492; 5.2 kcal/g, 60 kcal% fat, 20 kcal% protein, 20 kcal% carbohydrate; Research Diets), and HFD-P (D09020505; same composition as HFD-S of fat, protein, and carbohydrate, but 69% of the lard was exchanged for menhaden oil; Research Diets). The diets were matched to have similar macronutrient sources except for fat. The composition of the diets is shown in Table 1 and is previously published in Svahn SL et al. [2,3].

### 2.2 Harvest and preparation of the spleen

The mice were anesthetized and drained of blood before the spleens were dissected. Thereafter the spleens were snap-frozen in liquid nitrogen and stored at -80 °C until the extraction of proteins.

### 2.3 Proteomic analysis for relative quantification using iTRAQ

The proteomic analysis was performed at the Proteomic Core Facility at the Sahlgrenska Academy, Gothenburg University. Samples were homogenized in 200 µl (8 M urea, 50 mM triethylammonium bicarbonate (TEAB) using the FastPrep®-24 instrument (MP Biomedicals, OH, USA). A volume of 100 µl lysis buffer (50 mM TEAB, 8 M Urea, 4% Chaps, 0.2% SDS, 5 mM EDTA, pH 8.5) was added and total protein concentration was determined with Pierce™ BCA Protein Assay (Thermo Scientific). Aliquots containing 100 μg from each sample were reduced and alkylated according to the manufacturer's instructions and using buffers supplied in the kit (AB Sciex, iTRAQ Reagents Multi (4)-Plex Kit). A reference sample consisting of an aliquot from all samples was included in every set. Samples were in-solution digested by the addition of trypsin (1:50, trypsin to protein ratio, Promega) overnight at 37 °C. The resulting peptide samples were labeled with the iTRAQ4plex reagents and the samples were pooled into four independent sets. The sets were fractionated into 22 fractions by Strong Cation Exchange Chromatography (ÄKTA-system, Amersham-Pharmacia) on a PolySULFOETHYL A™ column (100 × 2.1 mm, 5 µm 300 Å, PolyLC inc.) over 40 minutes (0–100% 500 mM ammonium formate, pH 2.8 in 20% ACN).

Each fraction was desalted using PepClean C18 spin columns (Thermo Fisher Scientific) according to the manufacturer's guidelines. Samples were analyzed on an LTQ-Orbitrap Velos mass spectrometer interfaced with an Easy-nLC (Thermo Fisher Scientific). Peptides were separated on a C18 analytical column (220 × 0.075 mm I.D, 3 μm Reprosil-Pur C18-AQ particles, Dr. Maisch, Germany.) over a 90 min gradient from 5% to 80% ACN in 0.2% formic acid. The MS scans were performed at the resolution 60,000 with a mass range of *m/z* 400–1800. MS/MS analysis was performed in a data-dependent mode at 7500 in resolution and *m/z* 120–2000, with the top ten most abundant doubly or multiply charged precursor ions in each MS scan selected for MS/MS fragmentation. A parent mass list was used during the analysis. Dynamic exclusion was set to 30 s.

### 2.4 Data analysis

For relative quantification, the MS raw data files for each iTRAQ set were merged in the search using Proteome Discoverer version 2.4 (Thermo Fisher Scientific). The database search was performed with the Mascot search engine (Matrix Science) against Mus musculus in SwissProt version July 2019 (Swiss Institute of Bioinformatics, Switzerland). The data were searched with MS peptide tolerance of 10 ppm and MS/MS tolerance for the identification of 100 mmu. Tryptic peptides were accepted with zero missed cleavage variable modifications of methionine oxidation, and fixed modifications of cysteine methylthiol and N-terminal iTRAQ4plex and lysine iTRAQ4plex were selected. Perculator was used for PSM validation with the strict false discovery rate (FDR) threshold of 1%. Identified proteins were filtered at 5% FDR and grouped by sharing the same sequences to minimize redundancy. The quantification was normalized on the total peptide amount. The quantification was normalized using the protein median. Only peptides unique for a given protein were considered for relative quantitation, excluding those common to other isoforms or proteins of the same family. The results were then exported into Excel for manual data interpretation.

The analysis resulted in the identification of 924 proteins that were present in all datasets (Supplementary file S1). Qlucore Omics Explorer 3.6 (Lund, Sweden https://qlucore.com) was used for statistical analysis using *t*-test two-groups comparison (HFD-S vs LFD; HFD-P vs LFD; HFD-P vs HFD-S). With a false discovery rate cut-off < 0.1, 20 differentially expressed proteins were identified when HFD-P was compared with HFD-S, and no specific proteins were differentially expressed when HFD-S was compared with LFD or HFD-P compared with LFD (Fig. 2). All the 20 proteins that are differentially expressed between HFD-S and HFD-P diet showed an inverse protein expression pattern between the two groups (Fig. 3). *p*-values, false discovery rates, and fold changes for these 20 proteins were reported in supplementary file S1.

Categorization of the Gene Ontology terms for all 20 differentially expressed proteins was obtained by the information available in the PANTHER database [4]. These proteins were annotated for their role in biological processes and molecular functions using Gene Ontology. The majority of the proteins were found to be associated with cellular processes (28%), biological regulation (20.5%), and binding (53%). Out of 20 differentially expressed proteins, 11 proteins were found to be involved in cellular processes and 8 proteins were involved in biological regulation and binding. The detailed Gene Ontology categorization, the proteins involved in each category, and specific major sub-categorization are provided in Fig. 4.

## Declaration of Competing Interest

The authors declare that they have no known competing for financial interests or personal relationships which have, or could be perceived to have, influenced the work reported in this article.

## Appendix Supplementary materials

Image, application 1

Image, application 2
